# Supplementary material for: Drug repurposing for aging research using model organisms
Source: Aging Cell. 2017 Jun 16;16(5):1006–15. doi: 10.1111/acel.12626 (PMC5595691; doi:10.1111/acel.12626)
Supplement: Supplementary file 7 — Data S1 Zip‐Archive of all report cards. [file ACEL-16-1006-s007.zip › RC_P01.pdf]

P01

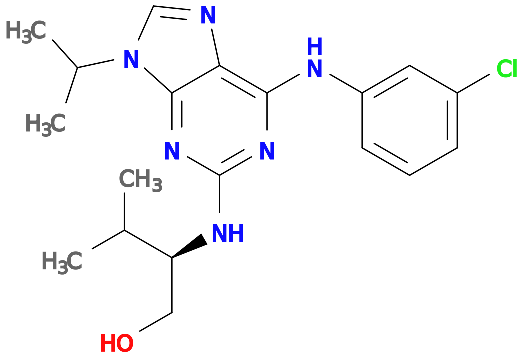

Database identifiers

ChEMBLCompound	CHEMBL23327
DrugBank	DB04751
CHEBI	47600
ZINC	ZINC00582575
eMolecules	593564

Ranking

	Rank	Score
Drosophila	7/697	0.948
C. elegans	73/591	0.366

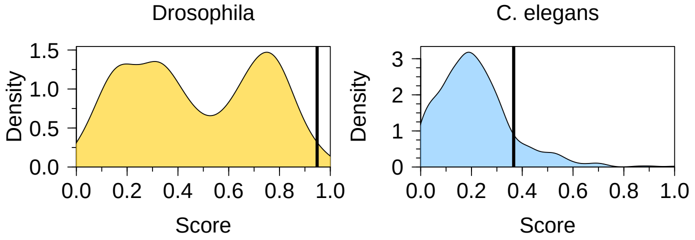

	Ageing implication	Domain conservation	Binding site conservation	Binding affinity	Bioavailability	Lipinski	Promiscuity	Purchasability	Drug approval	**Total**
Drosophila	1.0	0.962	1.0	0.894	(0.9)	0.0	-0.0	0.1	0.075	0.948
C. elegans	1.0	0.968	1.0	0.894	0.221	0.0	-0.0	0.1	0.075	0.366

Names

- **purvalanol A**
- 2-(1R-Isopropyl-2-hydroxyethylamino)-6-(3-chloroanilino)-9-isopropylpurine
- Piceatannol
- Purv

Roles

ChEBI entry 47600 has no roles

Status

Approved drug (according to ChEMBL)	No
Classification (according to DrugBank)	experimental
Number of Rule of 5 violations	0
Binding affinity to original target in log units (RF-Score prediction)	7.13
Burns *C. elegans* bioavailability prediction	-4.08

Compound Target Characteristics

Ribosomal protein S6 kinase alpha-1

Best gene implication in ageing for this target family came from gene Q86NP0 annotated in UniProt release 2014_02. Annotation GO 8340 (determination of adult lifespan) was Inferred from Mutant Phenotype

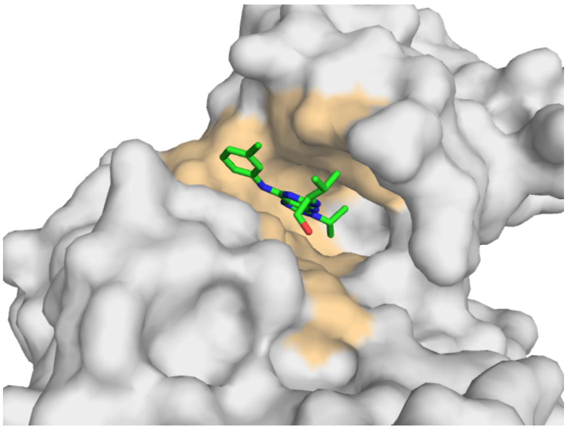

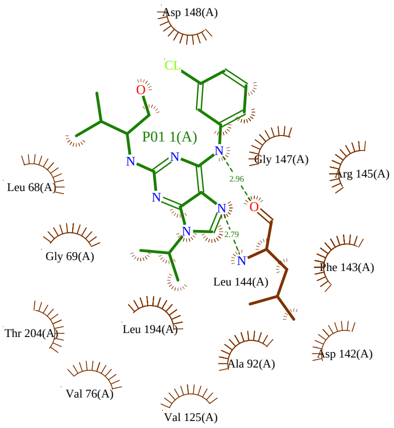

protein	amino acids contacts (binding site)
PDB:2z7s:chainA:Q15418	L G V A V D F L R G D E L T
tr:B1AXG1:B1AXG1_HUMAN	L G V A V D F L R G D E L T
tr:B7Z2K7:B7Z2K7_HUMAN	L G V A V D F L R G D E L T
tr:B7ZB17:B7ZB17_HUMAN	L G V A V D F L R G D E L T
tr:B4DG22:B4DG22_HUMAN	L G V A V D F L R G D E L T
tr:E9PRI4:E9PRI4_HUMAN	L G V A V D F L R G D E L T
tr:F5GYC4:F5GYC4_HUMAN	L G V A V D F L R G D E L T
tr:E9PGT3:E9PGT3_HUMAN	L G V A V D F L R G D E L T
sp:Q15418:KS6A1_HUMAN	L G V A V D F L R G D E L T
sp:Q15349:KS6A2_HUMAN	L G V A V D F L R G D E L T
tr:B7ZL90:B7ZL90_HUMAN	L G V A V D F L R G D E L T
sp:P51812:KS6A3_HUMAN	L G V A V D F L R G D E L T
sp:Q9UK32:KS6A6_HUMAN	L G V A V D F L R G D E L T
tr:F2Z2J1:F2Z2J1_HUMAN	L G V A V D F L R G D E L T
tr:F1M7N7:F1M7N7_RAT	L G V A V D F L R G D E L T
tr:F1LXV0:F1LXV0_RAT	L G V A V D F L R G D E L T
tr:D3Z8E0:D3Z8E0_RAT	L G V A V D F L R G D E L T
tr:F1LYL8:F1LYL8_RAT	L G V A V D F L R G D E L T
tr:B1AXP0:B1AXP0_MOUSE	L G V A V D F L R G D E L T
tr:Q810Z3:Q810Z3_MOUSE	L G V A V D F L R G D E L T
tr:B1AXN9:B1AXN9_MOUSE	L G V A V D F L R G D E L T
tr:E9PWV3:E9PWV3_MOUSE	L G V A V D F L R G D E L T
sp:P18653:KS6A1_MOUSE	L G V A V D F L R G D E L T
sp:Q9WUT3:KS6A2_MOUSE	L G V A V D F L R G D E L T
tr:F6RQA2:F6RQA2_MOUSE	L G V A V D F L R G D E L T
tr:A2CEE6:A2CEE6_MOUSE	L G V A V D F L R G D E L T
tr:Q505N6:Q505N6_MOUSE	L G V A V D F L R G D E L T
tr:F6Q8A4:F6Q8A4_MOUSE	L G V A V D F L R G D E L T
sp:P18654:KS6A3_MOUSE	L G V A V D F L R G D E L T
tr:A2CEE5:A2CEE5_MOUSE	L G V A V D F L R G D E L T
tr:Q810V8:Q810V8_MOUSE	L G V A V D F L R G D E L T
tr:E9PUP0:E9PUP0_MOUSE	L G V A V D F L R G D E L T
tr:A2CEE7:A2CEE7_MOUSE	L G V A V D F L R G D E L T
tr:E9QK39:E9QK39_MOUSE	L G V A V D F L R G D E L T
tr:Q86NP0:Q86NP0_DROME	L G V A V D F L R G D E L T
tr:Q9VR61:Q9VR61_DROME	L G V A V D F L R G D E L T
tr:Q24496:Q24496_DROME	L G V A V D F L R G D E L T
sp:Q21734:KS6A1_CAEEL	L G V A V D F L R G D E L T
tr:G5EE78:G5EE78_CAEEL	L G V A V D F L R G D E L T

protein	whole protein ident	simil	domain-based ident	simil	contact-based ident	simil
PDB:2z7s:chainA:Q15418	1.0	1.0	1.0	1.0	1.0	1.0
tr:B1AXG1:B1AXG1_HUMAN	0.25	0.28	0.65	0.7	1.0	1.0
tr:B7Z2K7:B7Z2K7_HUMAN	0.79	0.8	1.0	1.0	1.0	1.0
tr:B7ZB17:B7ZB17_HUMAN	0.79	0.91	0.9	0.97	1.0	1.0
tr:B4DG22:B4DG22_HUMAN	0.79	0.91	0.9	0.97	1.0	1.0
tr:E9PRI4:E9PRI4_HUMAN	0.96	0.97	1.0	1.0	1.0	1.0
tr:F5GYC4:F5GYC4_HUMAN	0.78	0.89	0.9	0.97	1.0	1.0
tr:E9PGT3:E9PGT3_HUMAN	0.98	0.98	0.96	0.96	1.0	1.0
sp:Q15418:KS6A1_HUMAN	1.0	1.0	1.0	1.0	1.0	1.0
sp:Q15349:KS6A2_HUMAN	0.77	0.92	0.89	0.97	1.0	1.0
tr:B7ZL90:B7ZL90_HUMAN	0.71	0.89	0.86	0.96	1.0	1.0
sp:P51812:KS6A3_HUMAN	0.76	0.88	0.9	0.97	1.0	1.0
sp:Q9UK32:KS6A6_HUMAN	0.71	0.88	0.86	0.96	1.0	1.0
tr:F2Z2J1:F2Z2J1_HUMAN	0.75	0.89	0.89	0.97	1.0	1.0
tr:F1M7N7:F1M7N7_RAT	0.77	0.92	0.89	0.97	1.0	1.0
tr:F1LXV0:F1LXV0_RAT	0.98	0.99	0.99	1.0	1.0	1.0
tr:D3Z8E0:D3Z8E0_RAT	0.77	0.88	0.9	0.97	1.0	1.0
tr:F1LYL8:F1LYL8_RAT	0.6	0.77	0.86	0.96	1.0	1.0
tr:B1AXP0:B1AXP0_MOUSE	0.24	0.26	0.6	0.64	1.0	1.0
tr:Q810Z3:Q810Z3_MOUSE	0.5	0.57	0.89	0.97	1.0	1.0
tr:B1AXN9:B1AXN9_MOUSE	0.79	0.91	0.9	0.97	1.0	1.0
tr:E9PWV3:E9PWV3_MOUSE	0.94	0.97	0.99	1.0	1.0	1.0
sp:P18653:KS6A1_MOUSE	0.97	0.98	0.95	0.96	1.0	1.0
sp:Q9WUT3:KS6A2_MOUSE	0.77	0.92	0.89	0.97	1.0	1.0
tr:F6RQA2:F6RQA2_MOUSE	0.93	0.96	0.99	1.0	1.0	1.0
tr:A2CEE6:A2CEE6_MOUSE	0.63	0.79	0.86	0.95	1.0	1.0
tr:Q505N6:Q505N6_MOUSE	0.98	1.0	0.99	1.0	1.0	1.0
tr:F6Q8A4:F6Q8A4_MOUSE	0.93	0.97	0.99	1.0	1.0	1.0
sp:P18654:KS6A3_MOUSE	0.77	0.88	0.9	0.97	1.0	1.0
tr:A2CEE5:A2CEE5_MOUSE	0.67	0.85	0.86	0.95	1.0	1.0
tr:Q810V8:Q810V8_MOUSE	0.94	0.95	0.99	1.0	1.0	1.0
tr:E9PUP0:E9PUP0_MOUSE	0.65	0.83	0.86	0.95	1.0	1.0
tr:A2CEE7:A2CEE7_MOUSE	0.66	0.84	0.86	0.95	1.0	1.0
tr:E9QK39:E9QK39_MOUSE	0.6	0.76	0.86	0.95	1.0	1.0
tr:Q86NP0:Q86NP0_DROME	0.45	0.67	0.76	0.92	1.0	1.0
tr:Q9VR61:Q9VR61_DROME	0.46	0.68	0.76	0.92	1.0	1.0
tr:Q24496:Q24496_DROME	0.46	0.68	0.76	0.92	1.0	1.0
sp:Q21734:KS6A1_CAEEL	0.55	0.8	0.82	0.94	1.0	1.0
tr:G5EE78:G5EE78_CAEEL	0.54	0.78	0.82	0.94	1.0	1.0

S6kII (FBgn0262866) associated phenotypes

dominant, learning defective, male limited, memory defective, neuroanatomy defective, reduced, short lived

(Information from FlyBase)

rskn-1 (UniProt:Q21734) annotation

Function: Serine/threonine kinase that may play a role in mediating the growth-factor and stress induced activation of transcription.
Cofactor:Mg(2+)
Enzyme regulation: Activated by multiple phosphorylations on threonine and serine residues.
Ptm: Autophosphorylated on Ser-422, as part of the activation process.

(Information from UniProt)

rskn-1 (UniProt:G5EE78) annotation

Cofactor: Mg(2+) Evidence=(PIRNR:PIRNR000606);

(Information from UniProt)
